# Fatal Disseminated Acanthamoeba lenticulata Acanthamebiasis in a Heart Transplant Patient

**DOI:** 10.3201/eid1305.061347

**Published:** 2007-05

**Authors:** Stéphane Barete, Alain Combes, Johan F. de Jonckheere, Annick Datry, Shaïda Varnous, Valérie Martinez, Sara García Ptacek, Eric Caumes, Frédérique Capron, Camille Francès, Claude Gibert, Olivier Chosidow

**Affiliations:** * Hôpital Tenon, Paris, France; † Hôpital Pitié-Salpêtrière, Paris, France; ‡ Scientific Institute of Public Health, Brussels, Belgium

**Keywords:** Heart transplantation, skin disease, infectious, skin ulcer, protozoan infections, Acanthamoeba, 18S ribosomal RNA, dispatch

## Abstract

We report a fatal case of disseminated acanthamebiasis caused by Acanthamoeba lenticulata (genotype T5) in a 39-year-old heart transplant recipient. The diagnosis was based on skin histopathologic results and confirmed by isolation of the ameba from involved skin and molecular analysis of a partial 18S rRNA gene sequence (DF3).

Acanthamoeba is 1 of 3 genera of free-living amebae that commonly cause disease in humans (*1*). These protozoa have been implicated in local infections, such as amebic keratitis, mainly in immunocompetent contact lens wearers, and in the mostly fatal, granulomatous amebic encephalitis in immunocompromised patients with HIV/AIDS, and immunosuppressant-treated patients, including organ transplant recipients (*2**–**4*). Disseminated acanthamebiasis (DA), which is defined as widespread extracerebral disease, is extremely rare, but its incidence has increased in recent years (*5*). Among DA reported, only 5 occurred in solid organ (3 lung and 2 kidney) transplant recipients (*4*). We report a fatal case of DA in a heart transplant recipient and identify Acanthamoeba lenticulata (genotype T5) as the cause of life-threatening disease.

## The Case

A 39-year-old man from Martinique had received a second heart transplant in March 2004 because of chronic rejection. He had received his first transplant 14 years earlier because of alcohol-related dilated cardiomyopathy. Skin complications included epidermoid carcinoma on the right leg in 1995 and diffuse viral warts on the trunk in 2003. Maintenance immunosuppression after the second heart transplant in 2004 included cyclosporine (220 mg/day), prednisone (20 mg/day), and mycophenolate mofetil (500 mg/day). The latter drug was withdrawn because of pancytopenia. Postsurgery complications included acute refractory bleeding (aortic anastomosis), cytomegalovirus infection of the gut, bacterial pulmonary infection, and postoperative renal failure that required chronic hemodialysis that prolonged his stay in the intensive care unit (ICU) to 5 months.

In January 2006, after a short visit to Martinique, the patient was transferred to our institution because of fever, dyspnea, and acute costal and back pain, with suspected osteitis underlying cutaneous lesions. Two months earlier, 4 trunk and leg abscesses or carbunclelike skin lesions has developed. Despite oral antistaphylococcal therapy, these lesions spread and became ulcerated and painful. Three ulcerated, violaceous plaques with undermined deep-infiltrated margins were present: 1 on the trunk (largest diameter 5 cm) (Figure, panel A) and 1 on each thigh. Three subcutaneous abscesses were present on the trunk and their puncture yielded a brown liquid. The differential diagnosis included pyoderma gangrenosum, neutrophilic dermatoses, mycobacteriosis, cutaneous bacterial infection, and calciphylaxis (chronic hemodialysis).

**Figure:**
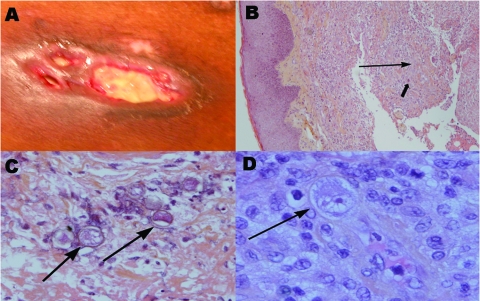
A) Ulcerated, violaceous plaque on the trunk of the patient with undermined infiltrated peripheral walls. B) Section of the lesion in A showing diffuse dermal-hypodermal necrosis with neutrophil infiltration (thin arrow) and sparse histiocytelike cells (thick arrow) (hematoxylin and eosin–stained, magnification ×10). C) Surgical skin biopsy specimen showing amebic cysts (arrows) in the dermal-hypodermal junction (hematoxylin and eosin–stained, magnification ×20). D) Surgical skin biopsy specimen showing intravascular amebic trophozoite (arrow) characterized by acanthopodia, cytoplasmic vacuoles, and a prominent nucleolus (hematoxylin and eosin–stained, magnification ×40).

The first histologic examination of a periulcerated skin lesion (punch biopsy specimen) showed diffuse dermal and hypodermal neutrophil infiltration and sparse histiocytelike cells (Figure, panel B). No infectious elements were identified. Biologic data indicated an inflammatory syndrome (C-reactive protein 250 mg/L [normal <5 mg/L], procalcitonin 25 ng/mL [normal <1 ng/mL]), with increased elevated circulating neutrophil counts (10.9 × 10^9^ cells/L) and anemia (hemoglobin 7 g/dL). Cultures of blood, abscess fluid, and involved skin were repeatedly negative for bacterial, mycotic, or parasitic agents. A computed tomographic body scan showed a massive abscess under the left kidney associated with pulmonary nodules without cutaneous calciphylaxis. Positron emission tomography scan confirmed those abnormalities and showed extensive and severe bone osteomyelitis.

Atypical pyoderma gangrenosum with visceral involvement was considered and treated with 3 intravenous prednisolone pulses. After minor initial improvement, the patient's condition deteriorated, and 10 days later septic shock associated with multiorgan failure developed. Surgical periulcer skin biopsy specimens were obtained in the ICU and specific parasite investigations were conducted. DA was then diagnosed. Hematoxylin and eosin staining of histologic sections showed cysts and trophozoites (30 μm diameter) in the dermal-hypodermal junction within polymorphous inflammatory granulomas associated with ischemic necrosis (Figure, panels C and D). The presence of Acanthamoeba sp. was confirmed by culturing amebae from involved skin on agar plates coated with Escherichia coli. In an indirect assay, the patient's serum showed weak immunofluorescence labeling against his own cultured cysts or vegetative amebae.

Molecular identification of DNA extracted from the isolated amebae was made by using the UNSET method (*6*). A diagnostic small subunit rDNA fragment (ASA.S1) was amplified by using JDP1 and JDP2 primers, and its differentiating fragment (DF3) was sequenced by using an internal 892c primer (*7*). The sequence of the DF3 subset that contains the highly variable and informative section in the ASA.S1 region of the Acanthamoeba isolate was visually compared with those of different published genotypes (T1–T15) (Table). Genotype T5 was identified (European Molecular Biology Laboratory accession no. AM411530). No drug-of-choice exists for treating DA. Despite treatment with pentamidine, 5-fluorocytosine, and itraconazole, the infection was rapidly fatal. Although analysis of cerebrospinal fluid obtained on days 5 and 18 after admission to the ICU showed no biochemical or parasite data suggestive of granulomatous amebic encephalitis, callus corpus necrosis was observed on a computed tomographic brain scan on day 18. The patient died of multiorgan failure on day 23. Family members refused to allow an autopsy.

**Table:** rDNA sequences of *Acanthamoeba* isolate (2/533) from the patient, a keratitis isolate (GAK1), 3 environmental T5 subtypes, and 4 other genotypes from persons with nonkeratitis infections

Genotype (strain)	DF3 sequence (5′→3′)*
T5 (2/533)	CAAAACACCGC**C**GTTAATCCTTT**TT**---CGGGGGTTAA**CG**GTTGGTGAAT
T5 (GAK1)	caaaacaccgc**c**gttaatccttt-----cgggggttaa**tg**gttggtgaat
T5 (72/2)†	caaaacaccgc**c**gttaatccttt-----cgggggttaa**tg**gttggtgaat
T5 (PD2S)‡	caaaacaccgc**t**gttaatccttt-----cgggggttaa**ta**gttggtgaat
T5 (FLAIV)§	caaaacaccgc**c**gttaatccttt**t**-**caa**cgggggttaacggttggtgaat
T4	caaaacacca-Atcggcgcggtcgtccttggcgtcggtccttcacggggccggcgcgagggcggcttagcccggtggcacc
T1	caaaacacca-ccatcaggcagtggggtcgtgcttcgcttttccggcaacggggaagtggaggcggtctcattcccctgatgg
T10	caaaacaccatccatttagcayggtcgttttcaaatattcctttttgcgaaggttgtttgggaacgattcgtcctgatggatc
T12	caaaacacca-ccattaacacgatcgttttttgcaaatatgccacatgcgcaagtgtgtggttgtgtttgaaggaacgatttg

*Sequences differences are shown in **boldface**. †Five isolates at European Molecular Biology Laboratory (EMBL). ‡Eight isolates at EMBL. §One isolate at EMBL.

## Conclusions

Protozoan infections are rare in heart transplant recipients, unlike in lung transplant recipients (*8**,**9*). To our knowledge, our patient, whose DA involved skin, bones, lungs, intraabdominal organs, and perhaps the brain, represents the first case to be reported in a heart transplant recipient. In a recent review of the literature, Duarte et al. (*4*) reported 5 cases of DA in lung (60%) or kidney (40%) transplant recipients. DA was difficult to diagnose in the patient, with 60% of the diagnoses made postmortem, which is similar to 74% of the diagnoses in 23 HIV/AIDS patients (*2*). The patient's clinical picture was atypical because his lesions were pyodermalike ulcers with subcutaneous abscesses, whereas the most frequently reported clinical skin manifestations were painful nodules, purpura, and pustules (*10*). Furthermore, the first histologic examination did not identify cysts. Acanthamoeba trophozoites with characteristic acanthopodia, cytoplasmic vacuoles, and a prominent nucleolus, especially in dermal vessels, were observed only after staining of the second biopsy specimen with hematoxylin and eosin in a context of strong clinical suspicion of DA. When reexamined retrospectively, the first skin biopsy specimen contained some pathogens, but trophozoites had been misidentified as histiocytelike cells.

Another important finding was the identification of the DA-causative agent as genotype T5, which is commonly found in the environment (*11*) and corresponds to A. lenticulata. This species has been isolated from nasal mucosa of persons without documented amebic infection (*12*). Although A. lenticulata has been shown to be pathogenic (*12*), genotype T5 was only recently isolated from a patient with keratitis (*13*). To our knowledge, our patient has the first case in which genotype T5 is the etiologic agent of a nonkeratitis, life-threatening DA infection.

Acanthamoeba spp. are free-living amebae found in soil, water, air, humans, and various animals (*14*). Depending on the molecular methods used (i.e., nuclear 18S rRNA or 16S rRNA mitochondrial gene amplification), 15 genotype sequences have been identified in environmental and human strains (T1–T15, Table). While genotype T4 is the most prevalent (79% of isolates) (*15*), only 1 A. lenticulata strain isolated from a patient with ocular keratitis had genotype T5 (*13*).

This case should alert physicians to a rare but life-threatening infection with A. lenticulata (genotype T5) in a heart transplant recipient. In organ transplant patients, when sterile cutaneous ulcers or subcutaneous abscesses develop that fail to respond to antibacterial treatments and pulse corticosteroids, histologic analysis should emphasize identifying Acanthamoeba spp.
